# Killing vector fields of locally rotationally symmetric Bianchi type V spacetime

**DOI:** 10.1038/s41598-024-58560-3

**Published:** 2024-05-03

**Authors:** Shakeel Ahmad, Tahir Hussain, Abdul Baseer Saqib, Muhammad Farhan, Muhammad Farooq

**Affiliations:** 1 Department of Basic Science and Humanities, CECOS University, Peshawar, Khyber Pakhtunkhwa Pakistan; 2 Department of Mathematics, University of Peshawar, Peshawar, Khyber Pakhtunkhwa Pakistan; 3 Faculty of Education, Nangrahar University, Jalalabad, Nangrahar Afghanistan

**Keywords:** LRS Bianchi type V metric, Killing symmetry, Rif tree approach, Kretschmann scalar, Mathematics and computing, Physics

## Abstract

The classification of locally rotationally symmetric Bianchi type V spacetime based on its killing vector fields is presented in this paper using an algebraic method. In this approach, a Maple algorithm is employed to transform the Killing’s equations into a reduced evolutive form. Subsequently, the integration of the Killing’s equations is carried out subject to the constraints provided by the algorithm. The algorithm demonstrates that there exist fifteen distinct metrics that could potentially possess Killing vector fields. Upon solving the Killing equations for all fifteen metrics, it is observed that seven out of the fifteen metrics exclusively exhibit the minimum number of Killing vector fields. The remaining eight metrics admit a varying number of Killing vector fields, specifically 6, 7, or 10. The Kretschmann scalar has been computed for each of the obtained metrics, revealing that all of them possess a finite Kretschmann scalar and thus exhibit regular behavior.

## Introduction

In 1916, Einstein introduced his theory of general relativity (GR), which completely transformed the traditional Newtonian notion of force by establishing a purely geometric understanding. As per the principles of GR, the idea of gravitational force is explained through the lens of curvature. In this framework, the presence of matter within the fabric of spacetime gives rise to curvature, which in turn governs the motion of test particles within that geometry. This theory formulates the dynamics of spacetime and associated concepts through a complex, non-linear system of equations, known as Einstein’s field equations (EFEs) given by^1^,

1.1 $$\begin{aligned} G_{pq} = R_{pq} - \frac{1}{2} R g_{pq} = kT_{pq}. \end{aligned}$$

Here, the components of Einstein tensor, Ricci tensor, stress-energy tensor and metric tensor are represented by $G_{pq}$, $R_{pq}$, $T_{pq}$ and $g_{pq}$ respectively, whereas *R* is Ricci scalar and *k* is coupling constant, which couples Einstein’s tensor and energy momentum tensor. In terms of physical interpretation, the EFEs provide a description of how the distribution of matter is interconnected with the presence of matter and energy within the spacetime. For $a, b = 0, 1, 2, 3,$ the EFEs result in a complex system of 10 interdependent, highly non-linear partial differential equations. The non-linear nature of these equations presents a significant challenge when it comes to determining their exact solutions^1^. The scarcity of physically significant exact solutions for these equations can be attributed to their non-linear nature, as only a few such solutions are found in the existing literature^1^. However, if we opt to define the arbitrary stress-energy tensor using the EFEs (1.1), it follows that any metric chosen will correspond to a solution of these equations. Nevertheless, it should be noted that not all solutions of this nature are meaningful since the corresponding energy-momentum tensor may exhibit highly non-physical characteristics. Hence, to obtain solutions that are physically relevant, certain restrictions would be expected to be fulfilled by the spacetime metric $g_{pq}$ representing those solutions. These constraints are formulated in relation to Killing vector fields (KVFs)^1^.

In the field of GR, various symmetries are extensively examined, which hold significant importance within the subject. Amongst these symmetries, the most fundamental one is characterized by a KVF. The presence of KVFs play a crucial role in the realm of spacetime physics as it preserves the spacetime metric and give rise to specific conservation laws^2^. In addition to KVFs, other notable symmetries in GR that are both well-known and of physical significance include Ricci collineations (RCs), which preserve the Ricci tensor; curvature collineations (CCs), which preserve the curvature tensor and matter collineations (MCs), which preserve the energy-momentum tensor. These symmetries are characterized by the condition that the Lie derivative of the metric, Ricci tensor, energy-momentum tensor, and Riemann tensor along the symmetry vector field is zero^2^. In simpler terms, a KVF corresponds to a smooth vector field that preserve the metric of a spacetime metric. Mathematically $\eta$ is said to be a KVF if,

1.2 $$\begin{aligned} \mathcal {L}_{\eta } g_{pq} = 0. \end{aligned}$$

Furthermore, it is worth noting that a Lie derivative can also preserve a spacetime metric, although with the possibility of introducing a conformal factor multiplied by the metric itself. This type of symmetry is characterized by a conformal Killing vector field (CKVF). Mathematically, a vector field $\eta$ is considered a CKVF if it fulfills the conformal Killing equation given by,

1.3 $$\begin{aligned} \mathcal {L}_{\eta } g_{pq} = 2\alpha (x^a)g_{pq}, \end{aligned}$$

where, $\alpha$ represents a real-valued function. The vector field $\eta$ transforms into a KVF when $\alpha$ in Eq. (1.3) is equal to zero. Furthermore, when $\alpha$ in Eq. (1.3) is a constant, the vector field $\eta$ defines a homothetic vector field. If we replace $g_{pq}$ with $R_{pq}$ in Eq. (1.3), it defines a conformal Ricci collineation, which reduces to an RC when $\alpha = 0$. If we consider the case when $g_{pq}$ is replaced by $R_{pq}$ and $\alpha$ is an arbitrary constant in Eq. (1.3), the vector field $\eta$ transforms into a homothetic Ricci collineation. Similarly, by replacing $g_{pq}$ with $T_{pq}$ in Eq. (1.3), we can define a conformal matter collineation. The primary objective of this paper is to examine and discuss Killing vector fields. As a result, our discussion will be centered exclusively on KVFs. KVFs can be identified as solutions to Killing’s Equation (1.2), which represents a system of 10 interconnected first-order partial differential equations. These equations are formulated in terms of four unknown functions that depend on four variables. When expressed in a coordinate system, the Killing equations take on a more convenient form, which can be stated as follows^2^,

1.4 $$\begin{aligned} \mathcal {L}_{\eta } g_{pq} = g_{pr} \eta ^r_{,q} + g_{rq} \eta ^r_{,p} + g_{pq,r} \eta ^r = 0 \end{aligned}$$

Within a 4-dimensional spacetime geometry, the maximum number of Killing vector fields is 10 when the spacetime metric is either flat or possesses constant curvature. Nevertheless, in the case of a non-flat spacetime geometry, it is anticipated that there will be less than or equal to seven Killing vector fields.

In order to emphasize the significance of Killing vector fields and their connections to other widely recognized symmetries, we provide a brief overview of recent literature. Bokhari et al. conducted a classification of spherically symmetric static spacetimes based on their Killing vector fields^3,4^. The authors, extended their research to explore the symmetry classification problem encompassing both RCs and CCs in the context of spherically symmetric spacetimes. Through their investigation, they established relationships between KVFs, RCs, and CCs^5,6^. In^7^, Moopanar and Maharaj explored conformal Killing vector fields of spherical spacetimes. A notable and extensive study conducted by Hussain et al. in 2019 focused on non-static spherically symmetric spacetimes and their conformal Ricci collineations^8^.

In 2003, Bokhari et al. extended the concept of Killing vector fields to provide a comprehensive classification of curvature collineations in cylindrically symmetric spacetimes^9,10^. Bokhari et al. extended their research on KVFs to explore matter collineations within a specific metric that exhibits static cylindrical symmetry^11^. In 2008, Bokhari et al. examined Killing symmetry of circularly symmetric static metric in three dimensions^12^. Feroze et al. carried out the classification of plane symmetric spacetimes by isometries^13^. Ziad presented the classification of static plane symmetric spacetime via their KVFs^14^. In 2004, Sharif investigated the symmetries of the energy-momentum tensor in static spacetimes with cylindrical symmetry^15^. In 2007, Shabbir et al. conducted a classification of static spacetimes with cylindrical symmetry based on their homothetic vector fields^16^. Ali and Feroze^17^ achieved a comprehensive classification of static spacetimes with cylindrical symmetry based on conservation laws.

In this paper, we have classified LRS Bianchi type V spacetimes according to their Killing vector fields using the Rif tree approach. This approach utilizes the Rif algorithm, which is an algorithmic framework developed using the Exterior package in the Maple plate form. The Rif algorithm consists of a set of commands that facilitate the classification process. This algorithm presents a comprehensive set of conditions that encompass all possible constraints on the metric functions. The graphical representation is portrayed as a tree, known as a Rif tree, where each branch represents specific conditions on the metric functions that determine whether the spacetime can possess Killing vector fields. Subsequently, one must solve the set of Killing’s equations, considering the conditions specified by the branches of the Rif tree, in order to obtain the explicit form of the Killing vector fields. Here we have obtained Killing algebras of dimension 4, 6, 7 and 10.

## Classification of LRS Bianchi type V spacetime

The metric of LRS Bianchi type is given as^18^:

2.1 $$\begin{aligned} ds^2=-dt^2+a(t)^2dx^2+e^{2mx}b(t)^2[dy^2+dz^2]. \end{aligned}$$

This metric possesses the following four minimum KVFs:

$$\begin{aligned} X_{(1)}= & {} \partial _y \\ X_{(2)}= & {} \partial _z \\ X_{(3)}= & {} z\partial _y-y\partial _z \\ X_{(4)}= & {} \partial _x-my\partial _y-mz\partial _z \end{aligned}$$

The coefficients of the above metric are used in the definition of KVFs to obtain the set of Killing equations given below:

2.2 $$\begin{aligned} \eta ^0_{,t}= & {} 0 \end{aligned}$$

2.3 $$\begin{aligned} \eta ^0_{,x}-a^2\eta ^1_{,t}= & {} 0 \end{aligned}$$

2.4 $$\begin{aligned} \eta ^0_{,y}-e^{2mx}b^2\eta ^2_{,t}= & {} 0 \end{aligned}$$

2.5 $$\begin{aligned} \eta ^0_{,z}-e^{2mx}b^2\eta ^3_{,t}= & {} 0 \end{aligned}$$

2.6 $$\begin{aligned} a^{\prime }\eta ^0+a\eta ^1_{,x}= & {} 0 \end{aligned}$$

2.7 $$\begin{aligned} a^2\eta ^1_{,y}+e^{2mx}b^2\eta ^2_{,x}= & {} 0 \end{aligned}$$

2.8 $$\begin{aligned} a^2\eta ^1_{,z}+e^{2mx}b^2\eta ^3_{,x}= & {} 0 \end{aligned}$$

2.9 $$\begin{aligned} b^{\prime }\eta ^0+mb\eta ^1+b\eta ^2_{,y}= & {} 0 \end{aligned}$$

2.10 $$\begin{aligned} \eta ^2_{,z}+\eta ^3_{,y}= & {} 0 \end{aligned}$$

2.11 $$\begin{aligned} b^{\prime }\eta ^0+mb\eta ^1+b\eta ^3_{,z}= & {} 0 \end{aligned}$$

**Figure 1:**
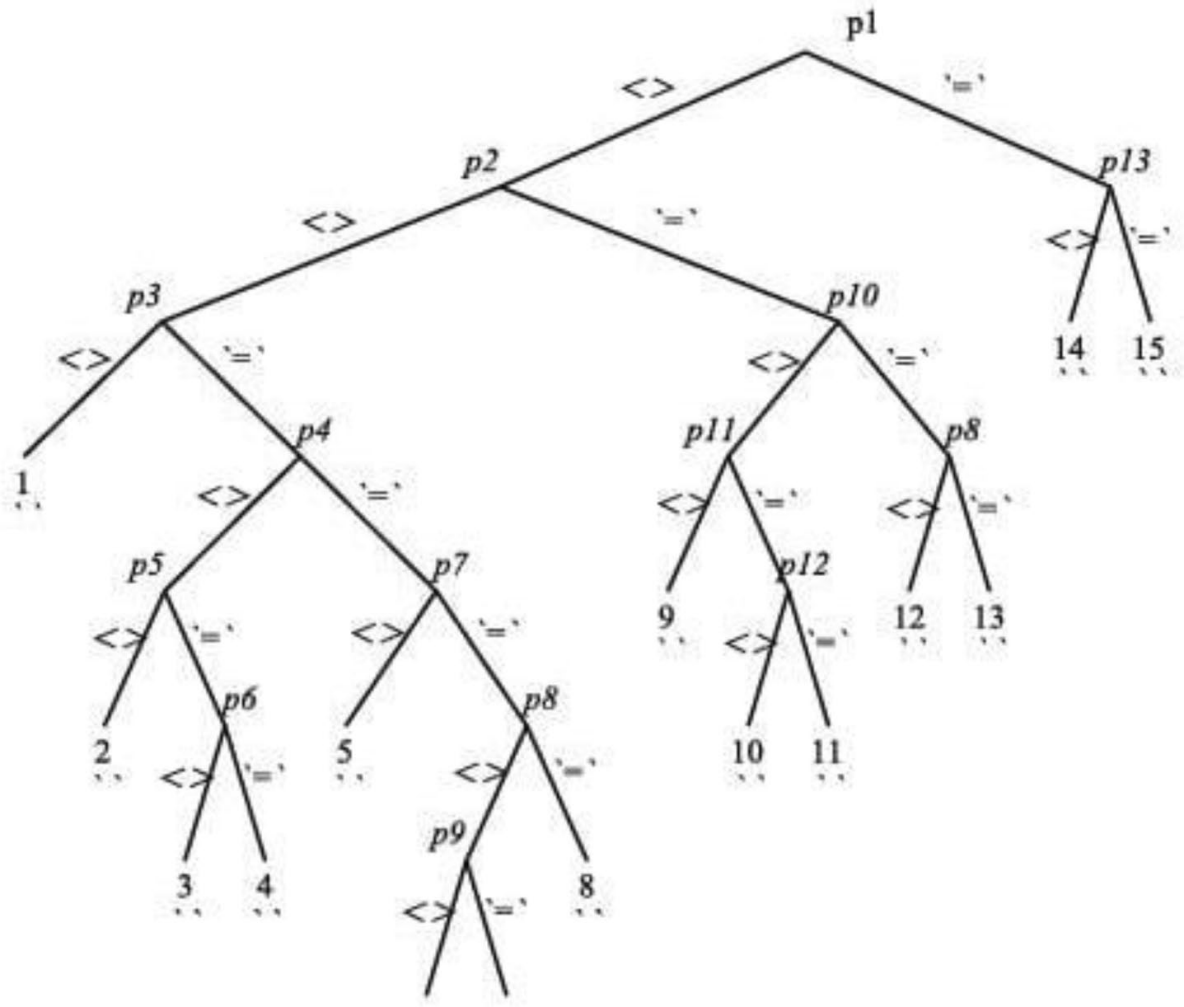
Rif Tree

In order to find the explicit form of KVFs, we need to solve these equations. Due to their non-linearity, these equations cannot be integrated directly without imposing some conditions on *a*(*t*) and *b*(*t*). For this purpose, we analyze these equations by Rif algorithm and as a result, we obtain the Rif tree given in Fig. 1. The expressions for nodes of the tree (pivots) are given in (2.12).

2.12 $$\begin{aligned} p_1&= b^{\prime }&p_2&= ba^{\prime }-b^{\prime }a \nonumber \\ p_3&= a^{\prime \prime }a^{\prime }-a^{\prime \prime \prime }a&p_4&= a^{\prime }[mb-b^{\prime }a][mb+b^{\prime }a] \nonumber \\ p_5&= m^2a^{\prime }b^3+b^{\prime \prime }b^{\prime }ba^3-(b^{\prime })^3a^3&p_6&= -m^2(a^{\prime })^2b^2-a^{\prime \prime }(b^{\prime })^2a^3+(a^{\prime })^2(b^\prime )^2a^2 \nonumber \\ p_7&= a^{\prime \prime }&p_8&= [mb-b^{\prime }a][mb+b^{\prime }a] \nonumber \\ p_9&= b^{\prime \prime }b-(b^{\prime })^2&p_{10}&= b^{\prime \prime } \nonumber \\ p_{11}&= b^{\prime \prime }b^{\prime }-b^{\prime \prime \prime }b&p_{12}&= m^2b^2+b^{\prime \prime }ba^2-(b^{\prime })^2a^2 \nonumber \\ p_{13}&= a^{\prime } \end{aligned}$$

Using the conditions of each branch, we have solved Eqs. (2.2)–(2.11) that yield Killing algebras of dimensions 4, 6, 7 and 10. In branches 1, 2, 3, 4, 5, 6 and 14, we have obtained only four KVFs. In the forthcoming sections, we summarize the results of remaining branches based on the dimension of the obtained Killing algebras.

### Six KVFs

This section contains metrics admitting six KVFs, four minimum and two additional ones. These metrics, along with their additional KVFs are presented in Tables 1 and 2.

### Seven KVFs

In this section, we present some metrics with seven KVFs. These metrics, along with their additional three KVFs are given in Table 3.

### Ten KVFs

There is only one metric, given by branch 13, that possesses ten KVFs. This metric is presented in Table 4 along with the solution of Killing equations and additional six KVFs.

**Table 1:** Metrics Admitting Six KVFs.

Branch No.	Metric	Solution of killing equations	Additional KVFs
9	$b^{\prime } \ne 0$, $ba^{\prime }-b^{\prime }a = 0$	$\eta ^0 = 0$, $\eta ^1 = -c_1 \frac{z}{m} +c_2 \frac{y}{m} + c_3$	$X_{(5)}=-\frac{z}{m}\partial _{x}+yz\partial _{y}$
	$b^{\prime \prime } \ne 0$ and $b^{\prime }b^{\prime \prime } - bb^{\prime \prime \prime } \ne 0$	$\eta ^2 = c_2 \bigg (\frac{z^2 - y^2}{2}+\frac{e^{-2mx}}{2m^2}\bigg ) +c_1yz$	$+ \big (\frac{z^2-y^2}{2} - \frac{e^{-2mx}}{2m^2} \big )\partial _{z}$
		$$+ c_4z - c_3my + c_5$$	
		$\eta ^3 = c_1 \bigg (\frac{z^2 - y^2}{2} - \frac{e^{-2mx}}{2m^2} \bigg ) - c_2yz - c_4y$	$X_{(6)} = \frac{y}{m}\partial _{x}$
		$- mc_3z + c_6$	$+ \big (\frac{z^2 - y^2}{2} + \frac{e^{-2mx}}{2m^2}\big )\partial _{y} - yz\partial _z$
10	$a(t) = b(t) = k_2 e^{k_1t} +k_3e^{-k_1t}$	$\eta ^0 =0$, $\eta ^1 = -c_1\frac{z}{m} +c_2\frac{y}{m} -\frac{c_3}{m}$	$X_{(5)}=-\frac{z}{m}\partial _{x}+yz\partial _{y}$
	where $k_1 \ne 0$ and	$\eta ^2 = c_2 \bigg (\frac{z^2 - y^2}{2} + \frac{e^{-2mx}}{2m^2}\bigg ) + c_1yz + c_4z$	$+ \big (\frac{z^2-y^2}{2} - \frac{e^{-2mx}}{2m^2} \big )\partial _{z}$
	$m^2 + 4k_1^2 k_2 k_3 \ne 0$	$+ c_3y + c_5$	
		$\eta ^3 = c_1 \bigg (\frac{z^2 - y^2}{2} - \frac{e^{-2mx}}{2m^2} \bigg ) - c_2yz - c_4y$	$X_{(6)} = \frac{y}{m}\partial _{x}$
		$+ c_3z + c_6$	$+ \big (\frac{z^2 - y^2}{2} + \frac{e^{-2mx}}{2m^2}\big )\partial _{y} - yz\partial _z$

**Table 2:** Metrics Admitting Six KVFs.

Branch No.	Metric	Solution of killing equations	Additional KVFs
11	$a(t) = b(t) = k_2 e^{k_1t} + k_3 e^{-k_1t}$	$\eta ^0 = 0$, $\eta ^1 = -c_1 \frac{z}{m} + c_2 \frac{y}{m} + c_3$	$X_{(5)}=-\frac{z}{m}\partial _{x}+yz\partial _{y}$
	where $k_1 \ne 0$ and	$\eta ^2 = c_2 \bigg (\frac{z^2 - y^2}{2} + \frac{e^{-2mx}}{2m^2}\bigg ) + c_1yz + c_4z$	$+ \big (\frac{z^2-y^2}{2} - \frac{e^{-2mx}}{2m^2} \big )\partial _{z}$
	$m^2 + 4k_1^2 k_2 k_3 = 0$	$- c_3my + c_5$	
		$\eta ^3 = c_1 \bigg (\frac{z^2 - y^2}{2} - \frac{e^{-2mx}}{2m^2}\bigg ) - c_2yz - c_4y$	$X_{(6)} = \frac{y}{m}\partial _{x}$
		$$ - c_3mz + c_6$$	$$+ \big (\frac{z^2 - y^2}{2} + \frac{e^{-2mx}}{2m^2}\big )\partial _{y} - yz\partial _z$$
12	$$a(t) = b(t) = k_1t + k_2 $$	$$\eta ^0 = 0$$, $$\eta ^1 = -c_1 \frac{z}{m} + c_2 \frac{y}{m} + c_3$$	$$X_{(5)}=-\frac{z}{m}\partial _{x}+yz\partial _{y} $$
	where $k_1 \ne 0$ and	$\eta ^2 = c_2 \bigg (\frac{z^2 - y^2}{2} + \frac{e^{-2mx}}{2m^2}\bigg ) + c_1yz + c_4z$	$+ \big (\frac{z^2-y^2}{2} - \frac{e^{-2mx}}{2m^2} \big )\partial _{z}$
	$m \ne \pm k_1$	$- c_3my + c_5$	
		$\eta ^3 = c_1 \bigg (\frac{z^2 - y^2}{2} - \frac{e^{-2mx}}{2m^2}\bigg ) - c_2yz - c_4y$	$X_{(6)} = \frac{y}{m}\partial _{x}$
		$$ - c_3mz + c_6$$	$$+ \big (\frac{z^2 - y^2}{2} + \frac{e^{-2mx}}{2m^2}\big )\partial _{y} - yz\partial _z$$

**Table 3:** Metrics Admitting Seven KVFs.

Branch No.	Metric	Solution of killing equations	Additional KVFs
7	$a(t) = k_1$ and $b(t) = e^{k_2t}$,	$\eta ^0 = \frac{c_1k_2z}{m^2-k_2^2} - \frac{c_2k_2y}{m^2-k_2^2} + c_3$	$X_{(5)}=\frac{k_2z}{m^2-k_2^2}\partial _t -\frac{mz}{m^2-k_2^2}\partial _x$
	where $k_1 \ne 0$, $k_2 \ne 0$	$\eta ^1 = -\frac{c_1mz}{m^2-k_2^2} + \frac{c_2my}{m^2-k_2^2} + c_4$	$+yz\partial _y - \frac{1}{2}\big [(y^2-z^2) + \frac{e^{-2(k_2t+mx)}}{m^2-k_2^2} \big ]\partial _z$
	and $m \ne \pm k_1k_2$	$\eta ^2 = \frac{c_2}{2}(z^2 - y^2) + c_1yz + c_5z - c_3k_2y$	$X_{(6)}= -\frac{k_2y}{m^2-k_2^2}\partial _t + \frac{my}{m^2-k_2^2}\partial _x$
		$- mc_4y + \frac{c_2e^{-2k_2t}e^{-2mx}}{2(m^2-k_2^2)} + c_6$	$- \frac{1}{2}\big [(y^2-z^2) -\frac{e^{-2(k_2t +mx)}}{m^2 -k_2^2} \big ]\partial _y - yz\partial _z$
		$\eta ^3 = -c_2yz + \frac{c_1}{2}(z^2-y^2) - c_5y$	$X_{(7)} = \partial _t - k_2y \partial _y - k_2z \partial _z$
		$$-\frac{c_1e^{-2k_2t}e^{-2mx}}{2(m^2-k_2^2)} - c_3k_2z - mc_4z + c_7 $$	
8	$a(t) = k_1t + k_2$ and $b(t) = a^{\frac{m}{k_1}}$,	$\eta ^0 = e^{k_1x} \bigg [c_1z + c_2y + c_3k_1\bigg ]$	$X_{(5)} = ze^{k_1x}\partial _t -\frac{ze^{k_1x}}{a}\partial _x +e^{(k_1-2m)x}\partial _z$
	where $m \ne k_1$ and $k_1 \ne 0$	$\eta ^1 = \frac{-e^{k_1x}}{a} \bigg [c_1z + c_2y + c_3k_1\bigg ] + c_4$	$X_{(6)} = ye^{k_1x}\partial _t -\frac{ye^{k_1x}}{a}\partial _x +e^{(k_1-2m)x}\partial _y$
		$\eta ^2 = c_5z + c_2 e^{(k_1 - 2m)x}\int \frac{dt}{b^2} - mc_4y + c_6$	$X_{(7)} = k_1e^{k_1x}\partial _t - \frac{k_1e^{k_1x}}{a}\partial _x$
		$$\eta ^3 = -c_5y + c_1 e^{(k_1 - 2m)x}\int \frac{dt}{b^2} - mc_4z + c_7 $$	
15	$$a(t) = $$ constant	$$\eta ^0 = c_1$$, $$\eta ^1 = \frac{c_2z}{m} - \frac{c_3y}{m} - \frac{c_4}{m}$$	$$X_{(5)} = \partial _t $$
	and $$b(t) = $$ constant	$$\eta ^2 = \frac{c_3}{2m^2}\big [-\frac{k_2^2}{k_1^2}e^{-2mx} - m^2y^2 + m^2z^2 \big ]$$	$$X_{(6)}=\frac{z}{m}\partial _x +yz\partial _y $$
		$+ c_2yz + c_4y + c_5z + c_6$	$+\frac{1}{2m^2}\big [ \frac{k_2^2}{k_1^2}e^{-2mx} - m^2y^2 + m^2z^2 \big ]\partial _z$
		$\eta ^3 = \frac{c_2}{2m^2}\big [\frac{k_2^2}{k_1^2}e^{-2mx} - m^2y^2 + m^2z^2 \big ]$	$X_{(7)} = -\frac{y}{m}\partial _x -\frac{1}{2m^2}\big [ \frac{k_2^2}{k_1^2}e^{-2mx}$
		$$- c_3yz + c_4z - c_5y + c_7 $$	$$ + m^2y^2 - m^2z^2 \big ]\partial _y -yz\partial _z $$

**Table 4:** Metric Admitting Ten KVFs.

Branch No.	Metric	Solution of killing equations	Additional KVFs
13	$a(t) = b(t) = mt + k_2$,	$\eta ^0 = e^{mx}\big [c_1m^2(y^2 + z^2) + c_2y + c_3z + c_4\big ]$	$X_{(5)}=\big [m^2e^{mx}(y^2+z^2)+e^{-mx} \big ]\partial _t$
	where $m \ne 0$	$+ c_1e^{-mx}$	$-\frac{1}{a}\big [m^2e^{mx}(y^2+z^2) -e^{-mx} \big ]\partial _x$
		$\eta ^1 = -e^{mx}\big [c_1m^2 \frac{y^2+z^2}{a(t)} +y\big (\frac{c_2}{a(t)} -\frac{c_5e^{-mx}}{m}\big )$	$-\frac{2m}{a}ye^{-mx}\partial _y -\frac{2m}{a}ze^{-mx} \partial _z$
		$+z\big (\frac{c_3}{a(t)} +\frac{c_6e^{-mx}}{m}\big ) + \frac{c_4}{a(t)}\big ] + \frac{c_1}{a(t)}e^{-mx} + c_7$	$X_{(6)}= ye^{mx}\partial _t -\frac{y}{a}e^{mx}\partial _x -\frac{e^{-mx}}{ma}\partial _y$
		$\eta ^2 = -\frac{2c_1me^{-mx}}{a(t)}y - \frac{c_2e^{-mx}}{ma(t)} + \frac{c_5}{2m^2}\big [e^{-2mx}$	$X_{(7)}=ze^{mx}\partial _t -\frac{z}{a}e^{mx}\partial _x -\frac{e^{-mx}}{ma}\partial _z$
		$- m^2y^2 + m^2z^2 \big ] + c_6yz - mc_7y + c_8z + c_9$	$X_{(8)} = \partial _t - \frac{e^{mx}}{a}\partial _x$
		$\eta ^3 = -\frac{2c_1me^{-mx}}{a(t)}z - \frac{c_3e^{-mx}}{ma(t)} - c_5yz + \frac{c_6}{2m^2}\big [-e^{-2mx}$	$X_{(9)} =\frac{y}{m}\partial _x +\frac{1}{2m^2}\big [e^{-2mx}$
		$- m^2y^2 + m^2z^2 \big ] - mc_7z - c_8y + c_{10}$	$-m^2y^2 + m^2z^2\big ] \partial _y -yz\partial _z$
			$$ X_{(10)} = -\frac{z}{m}\partial _x + yz\partial _y $$
			$$ - \frac{1}{2m^2}\big [ e^{-2mx} + m^2y^2 - m^2z^2 \big ]\partial _z $$

## Summary and discussion

In this paper, Killing vector fields have been calculated for LRS Bianchi type V spacetime. First we have used the metric of this spacetime in the definition of KVFs in order to obtain the set of ten Killing equations. A Maple algorithm, known as Rif algorithm was used to obtain a Rif tree along with the restrictions on the metric functions *a*(*t*) and *b*(*t*) under which the system of Killing equations has a solution. The algorithm gives fifteen different metrics which admit Killing vector fields of different dimensions. Solving Killing equations for all these metrics, we have observed that these metrics posses 4, 6, 7 or 10 dimensional Killing algebras. The Lie algebra for all the metrics possessing six KVFs is given by $[X_1,X_3]=X_2$, $[X_1,X_4]=-X_1$, $[X_1,X_5]=X_3$, $[X_1,X_6]=X_4,$ $[X_3,X_5]=X_6$, $[X_3,X_6]=-X_5$, $[X_4,X_5]=-X_5$, $[X_4,X_6]=-X_6,$ $[X_5,X_6]=-X_5.$ Similarly, the Lie algebra for the metric admitting ten KVFs is obtained as $[X_1,X_5]=X_6$, $[X_1,X_6]=X_8$, $[X_1,X_9]=X_4$, $[X_1,X_{10}]=X_3,$ $[X_2,X_5]=X_8$, $[X_2,X_7]=X_8$, $[X_2,X_9]=X_3$, $[X_2,X_{10}]=-X_4,$ $[X_3,X_5]=X_3$, $[X_3,X_6]=X_7$, $[X_3,X_7]=-X_6$, $[X_3,X_9]=-X_{10},$ $[X_3,X_{10}]=X_9$,$[X_4,X_9]=-X_9$,$[X_3,X_{10}]=-X_{10}$,$[X_5,X_6]=-X_9,$ $[X_5,X_7]=-X_{10}$, $[X_5,X_8]=X_5$, $[X_5,X_8]=X_5$,$[X_5,X_{10}]=X_5,$ $[X_6,X_7]=-X_3$, $[X_6,X_9]=X_{5}$, $[X_7,X_{10}]=X_{5}.$

The structure of Lie algebra for the metrics with seven KVFs is different from each other and it is presented in the following table:

**Table:** 

Branch	Lie algebra of metrics with seven KVFs
7	$[X_1,X_3]=-X_2$, $[X_1,X_4]=-X_1$, $[X_1,X_6]=X_7$, $[X_1,X_7]=-X_1,$ $[X_2,X_3]=X_1$,
	$[X_2,X_4]=-X_2$, $[X_2,X_6]=X_3$, $[X_2,X_7]=-X_2,$ $[X_3,X_5]=X_6$, $[X_3,X_6]=X_5$,
	$[X_4,X_5]=X_5$, $[X_4,X_6]=X_7,$ $[X_5,X_6]=X_5$, $[X_5,X_7]=X_5$, $[X_6,X_7]=X_7$
8	$[X_1,X_6]=X_7$, $[X_2,X_5]=X_7$, $[X_3,X_5]=X_6$, $[X_3,X_6]=X_5,$ $[X_4,X_5]=X_5$,
	$[X_4,X_6]=X_6$, $[X_4,X_7]=X_7$, $[X_5,X_6]=X_3,$ $[X_5,X_7]=X_2$, $[X_6,X_7]=X_1$
15	$[X_1,X_6]=X_3$, $[X_1,X_7]=X_4$, $[X_2,X_6]=X_4$, $[X_2,X_7]=X_3,$ $[X_3,X_6]=X_7$,
	$[X_3,X_7]=X_6$, $[X_4,X_6]=-X_6$, $[X_4,X_7]=-X_7,$ $[X_6,X_7]=X_3$

In order to add some physical implications, we find the source of matter for the obtained metrics and discuss their physical significance. It is well known that different forms of energy momentum tensor correspond to different matters. For example, $T_{ab}$ for a perfect fluid is of the form $T_{ab}=(\rho +p)u_au_b+pg_{ab},$ where $\rho$, *p* and $u_a$ signifies energy density, pressure and the four velocity vector respectively. The four velocity vector $u_a$ for the LRS Bianchi type V metric gets the form $u_a=(1,0,0,0)$ and hence $T_{ab}$ gives a perfect fluid if the non-diagonal component $T_{01}$ of energy-momentum tensor vanishes. Thus the metrics given by branches 8, 11, 12, 13 and 15 during our classification represent perfect fluid. For metrics of branches 8 and 13, the density and pressure become zero, giving vacuum solutions. The metric of branch 11 yields $\rho =3k_1^2$ and $p=-3k_1^2.$ As $\rho >0,$ it shows that the metric is physically realistic. Moreover, the values of $\rho$ and *p* satisfy all the energy conditions except the strong energy condition. For the metric of branch 12, the values of $\rho$ and *p* are obtained as $\rho =\frac{3(k_1^2-m^2)}{(k_1t+k_2)^2}$ and $p=-\frac{k_1^2-m^2}{(k_1t+k_2)^2}$. Here $\rho >0$ provided that $k_1^2 > m^2$ and under this condition all the energy conditions are identically satisfied. Finally, for the metric obtained in branch 15, we have $\rho =-\frac{3m^2}{k^2}$, $p=\frac{m^2}{k^2}$. As $\rho <0$, so this metric is an unrealistic metric.

In addition to the physical significance of the metrics discussed above, we also discuss the singularity of the obtained metrics. For this purpose, we calculate the Kretschmann scalar $K = R^{abcd}R_{abcd}$ for all the obtained metrics, where $R^{abcd}$ is the Riemann tensor. A spacetime is said to be regular (non-singular) when its associated Kretschmann scalar possesses a finite value. Here, we check the singularity of the metrics which have more than four KVFs.

For the metric of branch 7, the obtained value of *K* is given by:

$$\begin{aligned} K = \frac{12}{k_1^4}\big (k_2^2 k_1^2 - m^2 \big )^2 \end{aligned}$$

Here the value of *K* is clearly finite and positive. So this metric has no singularity.

The Kretschmann scalar *K* becomes zero for the metrics associated with branches 8 and 13, indicating that these two metrics exhibit regularity.

The metric associated with branch 9 yields the value of *K* as:

$$\begin{aligned} K = \frac{12}{a(t)^4} \big [\big (m^2 - a^{{\prime }2} \big )^2 + a^2 a^{{\prime \prime }2} \big ] \end{aligned}$$

Similar to the branch 7 metric, the metric of this branch is also regular, featuring a positive (finite) Kretschmann scalar.

The Kretschmann scalar *K* for the metrics corresponding to branches 10 and 11 is expressed as:

$$\begin{aligned} K = \frac{12}{(k_1t + k_2)^4} \big [\big (m^2 - k_1^2 \big (k_2e^{k_1t} - k_3e^{-k_1t} \big )^2 \big )^2 + k_1^4 \big (k_2e^{k_1t} + k_3e^{-k_1t} \big )^4\big ]. \end{aligned}$$

It can be seen that the metrics of these branches are regular, having finite value of *K*.

In case of branch 12, the value of *K* is determined to be:

$$\begin{aligned} K = \frac{12}{(k_1t + k_2)^4} \big (m^2 - k_1^2 \big )^2, \end{aligned}$$

which is evidently positive and finite, indicating a metric that is regular.

The value of *K* for branch 15 gets the form:

$$\begin{aligned} K = \frac{12 m^4}{\xi ^4}, \end{aligned}$$

that demonstrates the absence of singularity in the metric.

## Data Availability

The data sets used and/or analyzed during the current study are available from the corresponding author on reasonable request.
